# Network Modeling Sex Differences in Brain Integrity and Metabolic Health

**DOI:** 10.3389/fnagi.2021.691691

**Published:** 2021-06-29

**Authors:** Janelle T. Foret, Maria Dekhtyar, James H. Cole, Drew D. Gourley, Marie Caillaud, Hirofumi Tanaka, Andreana P. Haley

**Affiliations:** ^1^Department of Psychology, The University of Texas at Austin, Austin, TX, United States; ^2^Department of Computer Science, Centre for Medical Image Computing, University College London, London, United Kingdom; ^3^Dementia Research Centre, Institute of Neurology, University College London, London, United Kingdom; ^4^Department of Kinesiology and Health Education, The University of Texas at Austin, Austin, TX, United States; ^5^Biomedical Imaging Center, The University of Texas at Austin, Austin, TX, United States

**Keywords:** sex differences, metabolic syndrome, network model, white matter hyper intensities, brain-predicted age, functional connectivity, APOE, magnetic resonance spectroscopy

## Abstract

Hypothesis-driven studies have demonstrated that sex moderates many of the relationships between brain health and cardiometabolic disease, which impacts risk for later-life cognitive decline. In the present study, we sought to further our understanding of the associations between multiple markers of brain integrity and cardiovascular risk in a midlife sample of 266 individuals by using network analysis, a technique specifically designed to examine complex associations among multiple systems at once. Separate network models were constructed for male and female participants to investigate sex differences in the biomarkers of interest, selected based on evidence linking them with risk for late-life cognitive decline: all components of metabolic syndrome (obesity, hypertension, dyslipidemia, and hyperglycemia); neuroimaging-derived brain-predicted age minus chronological age; ratio of white matter hyperintensities to whole brain volume; seed-based resting state functional connectivity in the Default Mode Network, and ratios of N-acetyl aspartate, glutamate and myo-inositol to creatine, measured through proton magnetic resonance spectroscopy. Males had a sparse network (87.2% edges = 0) relative to females (69.2% edges = 0), indicating fewer relationships between measures of cardiometabolic risk and brain integrity. The edges in the female network provide meaningful information about potential mechanisms between brain integrity and cardiometabolic health. Additionally, Apolipoprotein ϵ4 (ApoE ϵ4) status and waist circumference emerged as central nodes in the female model. Our study demonstrates that network analysis is a promising technique for examining relationships between risk factors for cognitive decline in a midlife population and that investigating sex differences may help optimize risk prediction and tailor individualized treatments in the future.

## Introduction

Sex has emerged as a moderator in many associations between brain health and cardiometabolic dysfunction. More specifically, sex appears to moderate associations between cognition and aortic stiffness (Sabra et al., 2020), between risk of dementia and plasma lipid and lipropoteins (Ancelin et al., 2013; Gilsanz et al., 2017), and between white matter hyperintensities and adiposity (Alqarni et al., 2021). Additionally, there are pronounced sex differences in cardiovascular aging, particularly in the structures and function of the vasculature (Merz and Cheng, 2016). For example, type 2 diabetes and elevated systolic blood pressure may result in slightly higher risk for cardiovascular disease for women (Wei et al., 2017) and greater cardiovascular burden for aging women than men (Huebschmann et al., 2019). There are also sex differences in important brain biomarkers, such as connectivity (Gong et al., 2011; Zhang et al., 2018) and age-related atrophy (Xu et al., 2000). In later life, women demonstrate higher rates of Alzheimer's Disease (AD), even when controlling for survivorship effects (Zhao et al., 2016; Andrew and Tierney, 2018; Beam et al., 2018; Buckley et al., 2019). Since cardioprotective effects of estrogen that provide advantage to women (Stanhewicz et al., 2018; Peters et al., 2019; Rodgers et al., 2019) end at menopause and later life cognitive declines may originate at midlife or earlier (Rodrigue et al., 2013; Irwin et al., 2018), age 40–60 is an opportune period for investigating relationships between brain and metabolic variables for males and females separately.

Growing evidence for the causal influence of multiple variables on biological systems has increased the need for new statistical techniques that can provide greater insight into complex relationships at once. Network analysis has been primarily applied to psychiatric comorbidity (Borsboom and Cramer, 2013), and the use in biological models is relatively novel. This technique provides a visual depiction of the complex associations among symptoms, which can be understood as partial correlations. Network analysis also allows identification of “central” symptoms, defined by strong correlations with a large number of other symptoms. The theory is that, similar to a domino effect, the presence of a central symptom is likely to have greater influence over the entire network of symptoms due to its high degree of interconnectedness (van Borkulo et al., 2015; Beard et al., 2016). In respect to networks with biological variables, centrality of a node in a network may convey that a variable has an impact on other variables or may drive relationships between variables. For the purposes of research in risk for cognitive decline, centrality can help untangle which metabolic risk factors have the greatest influence on brain integrity for males vs. females.

Through two separate exploratory network analyses for males and females, we sought to understand sex differences in the relationships between brain integrity and metabolic risk. Our variables of interest, markers of brain integrity, age, genetic status and the components of metabolic syndrome, were selected based on evidence linking them to late-life cognitive decline. We hypothesized that there would be sex differences in these networks, but we did not form specific hypotheses about relationships between variables, other than anticipating that higher levels of metabolic risk factors would be more likely to relate to poorer brain integrity for both males and females.

## Materials and Methods

### Variables of Interest

#### Metabolic Syndrome Components

Metabolic Syndrome (MetS) and its five key components have been established as a cluster of risk factors for cardiovascular disease, which include: abdominal obesity, high triglyceride concentrations, low high-density lipoprotein (HDL) cholesterol, above normal blood pressure (prehypertension), and above normal blood sugar (prediabetes) (Eckel et al., 2005). MetS diagnosis is indicated by meeting criteria for 3 or more components based on the Alberti et al. (2009) consensus criteria to determine cut offs for each MetS category: fasting glucose ≥100 mg/dL or treatment for hyperglycemia, triglycerides ≥150 mg/dL, HDL-cholesterol ≤ 40 mg/dL in males and ≤ 50 mg/dL in females or treatment for dyslipidemia, systolic blood pressure ≥130 mmHg or diastolic ≥85 mmHg or antihypertensive medication, and waist circumference ≥102 cm for men and ≥88 cm for women. MetS category variables were coded as yes or no to indicate whether or not an individual met criteria for each variable of interest. MetS diagnosis and key components have been associated with negative cognitive consequences (Skoog et al., 1996; Waldstein et al., 2004; Yaffe et al., 2004; Kivipelto et al., 2005; Whitmer et al., 2005; Segura et al., 2009; Arvanitakis et al., 2010; Falkowski et al., 2014; Foret et al., 2020b). Sex differences have been observed in MetS, such as differences in prevalence and age at incidence (Regitz-Zagrosek et al., 2007; Yang and Kozloski, 2011) and clustering of risk factors (Kuk and Ardern, 2010).

#### Brain-Predicted Age Difference (Brain-PAD)

Machine-learning methods that measure biological aging can aid in early detection of brain vulnerability (Cole et al., 2019) and serve as important predictors of mortality (Horvath, 2013; Putin et al., 2016; Cole et al., 2018). One such method, neuroimaging-derived ‘brain age', estimates an individual's biological age based on gray and white matter volumes (Cole, 2017; Cole and Franke, 2017). By subtracting chronological age from brain age (brain-PAD), it is possible to estimate which individuals might have poorer brain health in terms of volumetric loss, which may relate to risk for neurocognitive decline. For example, in a sample of individuals with Down's Syndrome, elevated brain-PAD has been linked to amyloid deposition and cognitive decline (Cole et al., 2019). We calculated the brain-PAD of individuals in our dataset to determine which individuals might have “older” brains than their chronological age, such that a higher, positive brain-PAD would reflect higher levels of atrophy.

#### White Matter Hyperintensities (WMH)

WMH are areas of hyperintense signal on MRI indicative of lesions in the deep white matter, produced through chronic hypoperfusion and disruption of the blood-brain barrier (van Swieten et al., 1991; Pantoni and Garcia, 1997; Debette and Markus, 2010; Topakian et al., 2010). WMH are commonly observed in aging populations but have been associated with vascular and metabolic risk even after correcting for age (Launer, 2003; Yoshita et al., 2006; Birdsill et al., 2014). Specific components of MetS have been associated with WMH and research has suggested white matter lesions as the mechanism behind cognitive decline in populations with MetS (Alfaro et al., 2016). Relationships between WMH and cardiometabolic risk at midlife have been observed in both cross-sectional (Pasha et al., 2017) and longitudinal follow-up studies (Aljondi et al., 2020). Additionally, relationships between WMH, cardiovascular risk factors, and later life cognitive decline are marked by sex differences (Pasha et al., 2018b; Burke et al., 2019; Alqarni et al., 2021).

#### Rs-fcMRI

Resting state functional connectivity MRI (rs-fcMRI) identifies temporal correlation of brain regions through low frequency background fluctuations in neuronal activity measured by the blood oxygen level dependent (BOLD) signal during a period of rest (Biswal et al., 1995; Fox and Greicius, 2010). *rs-*fcMRI is one way to examine early brain vulnerability, particularly through examining one of the most widely-studied networks in this context, the Default Mode Network (DMN). The primary nodes of the DMN are the dorsal and ventral medial prefrontal cortices (dMPFC and vMPFC) and the posterior cingulate cortex (PCC) (Greicius et al., 2003). Both the MPFC and PCC have relationships to age-related brain pathologies (Zhou et al., 2008, 2016; Zhang et al., 2010). Dyssynchrony in the DMN is thought to occur before clinical manifestation of the disease and changes in structure (Habib et al., 2017) and may be one of the earliest markers for late-life cognitive decline. Additionally, differential relationships between DMN connectivity and executive function have been examined in middle-aged adults with varying numbers of MetS components (Foret et al., 2020a). While sex differences have been observed in task-based functional connectivity at midlife (Jacobs et al., 2017), relationships between sex and DMN dyssychrony requires further investigation.

#### Magnetic Resonance Spectroscopy (^1^H MRS)

Proton Magnetic Resonance Spectroscopy allows for detection of cerebral metabolites. ^1^H MRS may have greater sensitivity to tissue vulnerability than MRI and thus is appropriate for early, pre-clinical changes in midlife brain metabolism (Barker et al., 1994). Three metabolites were selected for their significance in neurobiological models of aging: N-acetyl aspartate (NAA), a metabolite that is highly concentrated in neurons and considered a marker of neuronal health (Danielsen and Ross, 1999; Haley et al., 2010b; Gonzales et al., 2013); glutamate, an excitatory neurotransmitter implicated in synaptic plasticity (Danielsen and Ross, 1999) and metabolic health (Haley et al., 2010a, 2012; Magi et al., 2019); and *myo*-inositol (mI), an organic osmolyte and substrate for the synthesis of the secondary messenger, inositol triphosphate, which has been elevated in beta amyloid positive individuals and associated with decreased DMN connectivity independent of amyloid accumulation (Voevodskaya et al., 2016, 2019). Significant sex differences have been observed in cerebral metabolites, particularly in concentrations of NAA and mI, as early as childhood and adolescence (Cichocka et al., 2018).

#### Apolipoprotein E (ApoE)

The allele frequency of ApoE ϵ4 (ϵ4) in the ApoE genotype has been consistently associated with increased risk for AD (Roses and Saunders, 1994; Green et al., 2009) and other forms of neurocognitive decline (Rohn, 2014; Mukerji et al., 2016). Disruptions in neuronal metabolism due to ApoE's role in cholesterol transport are cited as the mechanism behind this association (Lahoz et al., 2001; Eichner et al., 2002). Young healthy ϵ4 carriers have distinct patterns of activity in the DMN (Filippini et al., 2009). Additionally, research has shown that the effect of ϵ4 status on Alzheimer's risk may be stronger for female than male carriers (Sampedro et al., 2015; Riedel et al., 2016).

#### Age

Many of the above risk factors and markers of neuropathology have the strongest relationships with cognitive decline as individuals age (Hädel et al., 2013; Vidal-Piñeiro et al., 2014; Makkar et al., 2020). However, the impact of age at midlife on the relationships between metabolic health and brain integrity is not fully understood. Including this variable in the model could provide further information about the importance of age in these relationships.

### Participants

Four hundred nine adults between the ages of 40 and 61 were recruited for the study through local newspaper advertisements and flyers. Among them, 274 individuals were enrolled, and metabolic, demographic and imaging data were available on 266 participants. Exclusion criteria were history of neurological disease, major psychiatric illness, history of substance abuse, or MRI contraindication.

When grouping by sex, there were no significant differences between groups in age [*t*_(264)_ = −0.19, *p* = 0.850], education [*t*_(258)_ = 1.41, *p* = 0.160], or ApoE status (χ^2^(1, *N* = 244) < 0.001, *p* = 1). Males had significantly higher levels of mI [*t*_(201)_ = 2.85, *p* < 0.01], waist circumference [*t*_(259)_ = 3.38, *p* < 0.001], triglycerides [*t*_(239)_ = 2.25, *p* = 0.025] and glucose [*t*_(260)_ = 3.13, *p* < 0.01] while females had significantly higher functional connectivity [*t*_(204)_ = −2.38, *p* = 0.018] and HDL-cholesterol [*t*_(254)_ = −7.08, *p* < 0.001]. Only 12 female participants were actively taking hormone replacement therapies (HRT), which was not a sufficient sample size to include HRT as a covariate in our analyses. Participant characteristics are provided in Table 1.

**Table 1:** Selected participant characteristics (*n* = 266).

**Participant characteristics**	**Male**			**Female**			
	***N***	***Mean ± SD***		***N***	***Mean ± SD***	***t***	***p***
Age, y	121	49 ± 6		145	49 ± 6	−0.19	0.850
Education, y	118	16 ± 3		142	16 ± 2	1.41	0.160
MMSE	116	29 ± 2		133	29 ± 2	−0.16	0.874
ApoE ϵ4, (yes/no)	118	103/15		126	109/17	χ^2^ <0.001	1
**Neuroimaging Measures**							
WMH/TIV	78	0.002 ± 0.002		82	0.002 ± 0.003	0.71	0.476
brain–PAD, years	92	−4.8 ± 6.7		110	−6.3 ± 6.8	1.49	0.137
NAA/Cre	90	1.34 ± 0.24		117	1.35 ± 0.22	−0.08	0.936
Glutamate/Cre	88	1.25 ± 0.15		115	1.23 ± 0.11	1.45	0.149
mI/Cre	88	0.77 ± 0.09		115	0.73 ± 0.08	2.85	0.005
DMPFCxPCC	88	0.18 ± 0.30		118	0.28 ± 0.28	−2.38	0.018
**Metabolic Measures**							
Systolic blood pressure, mmHg	118	138 ± 22		144	136 ± 22	0.66	0.508
Waist circumference, cm	118	101 ± 15		143	94 ± 16	−3.51	<0.001
HDL–cholesterol, mg/dL	117	42 ± 15		139	56 ± 16	2.97	0.003
Triglyceride, mg/dL	109	128 ± 68		133	109 ± 63	2.25	0.025
Blood glucose, mg/dL	118	104 ± 32		142	94 ± 22	3.50	<0.001
Physical activity, hours/week	117	1.66 ± 2.15		140	1.42 ± 1.60	1.02	0.311
**Mets Criteria**							
Systolic blood pressure, (yes/no)	118	16%/84%		144	13%/87%		
Waist circumference, (yes/no)	118	47%/53%		143	69%/31%		
HDL-cholesterol, (yes/no)	117	50%/50%		139	32%/68%		
Triglyceride, (yes/no)	109	47%/53%		133	27%/73%		
Blood glucose, (yes/no)	118	43%/57%		142	23%/77%		

*Brain-PAD, Brain predicted age difference; DMNPFCxPCC, Resting State Functional Connectivity in the Default Mode Network; NAA, N-Acetylaspartate; mI, Myo-inositol; Cre, Creatine; WMH/TIV, White Matter Hyperintensities adjusted for total intracranial volume; Physical Activity sum of hours moderate*.

### Procedures

The Institutional Review Board at the University of Texas at Austin approved all study procedures. Written informed consent before enrolling in the study was provided by participants. Medical history was collected through self-report questionnaires and participants underwent a neuropsychological evaluation, brain imaging and a general health assessment. Assessments and imaging were completed in separate visits and most participants completed the study in 1 month.

### Neuropsychological Assessment

Participants completed a neuropsychological battery consisting of tests of memory, verbal fluency and executive function. Raw scores from a neuropsychological battery were converted to sample-based z scores. Scores from the Mini-Mental Status Exam (MMSE; Kurlowicz and Wallace, 1999); the California Verbal Learning Test-2nd Edition, short delay free recall, long delay free recall and recognition discriminability conditions (CVLT-II; Delis et al., 2000); Digit Span forward and backward conditions total score from the Weschler Adult Intelligence Scale—Fourth Edition (WAIS-IV) (Lichtenberger and Kaufman, 2012); Controlled Oral Word Fluency total score (Ruff et al., 1996); Stroop Color and Word Test, third condition (Jensen and Rohwer, 1966); and inverted scores from the Trail Making Test (Bowie and Harvey, 2006), conditions A and B were combined into an average overall current cognitive test performance score, to limit the number of comparisons.

### Health Assessment

Blood samples were collected after 8 h of fasting using venipuncture of the antecubital vein and resting blood pressure was measured with a semiautomated device following 15 min of rest (VP-1000, Omron Healthcare, Bannockburn, IL). A non-elastic tape measure was used for waist and hip circumference. Blood concentrations of glucose, triglycerides, total cholesterol and HDL-cholesterol were measured using a standard enzymatic technique. Participants were asked to report hours per week of low, moderate (e.g., fast walking, tennis, easy bicycling, easy swimming) or vigorous (e.g., running, jogging, hockey, vigorous swimming) physical activity which exceeded 15 min intervals, based on the classifications used by the Godin leisure-time physical activity questionnaire (Godin and Shephard, 1985). Hours of moderate and vigorous physical activity were summed to derive a total measure of weekly physical activity (Pasha et al., 2018a).

Saliva samples were collected using the Oragene Discover (OGR-500) kit and stored at room temperature prior to analysis. The prepIT·L2P kit from DNAgenotek was used for DNA extraction using 500 μL of saliva. Samples were stored at −40°C prior to genotyping. ApoE-Fwd4 and ApoE-snapR primers were used for polymerase chain reaction amplification, which was performed with 10 ng of DNA and 10 pMol primer. Amplification protocol was as follows: 95°C for 15 min, 35 cycles of (95°C 30 s, 65°C 30 s, 72°C 30 s) and hold at 4°C.

ApoE genotyping was conducted using PCR amplification and Sanger sequencing (Sanger et al., 1977) with Variant Reporter Software from Life Technologies (Thermo Fisher Scientific). Sequence data was obtained with KB basecaller and chromatograms were analyzed via visual inspection for the rs429358C>T and rs7412C>T SNPs. Participants were categorized according to allele type. Due to sample size, ApoE ϵ4 hetero- and homozygous individuals were combined together (*n*_*male*_ = 15; *n*_*female*_ = 17), and compared with all ApoE ϵ4 non-carriers (*n*_*male*_ = 103; *n*_*female*_ = 109).

### MRI Data

#### Structural MRI

Structural images were collected, registered, and normalized to MNI space. The entire brain was included in structural images and were collected in the sagittal plane using a high-resolution magnetization prepared rapid gradient echo (MPRAGE) sequence (256 × 256 matrix, flip angle = 7°, FOV = 24 × 24 cm^2^, 1 mm slice thickness, 0 gap).

Brain-predicted age was estimated using the machine-learning framework (Gaussian Process) devised by Cole (2017) and Cole et al. (2017) and trained on data available via public repositories from 2001 healthy individuals ages 18–90. brain-PAD was calculated by subtracting chronological age from brain-predicted age.

WMH volume was quantified by Lesion Segmentation Tool version 1.2.3 (http://www.applied-statistics.de/lst.html), which is an automated algorithm implemented in SPM8 (http://www.fil.ion.ucl.ac.uk/spm/software/spm8/). As previously described by Pasha et al. (2017), voxels were assigned to tissue probability maps and given a probability of being a white matter lesion based on spatial and intensity probabilities from T1 images and hyperintensity outliers on T2 FLAIR images. An initial threshold of 0.30 was applied to a conservative lesion belief map to create lesion seeds. A growth algorithm then grew these seeds toward a liberal lesion belief map and a final threshold of 0.99 was applied to the resulting lesion belief map to remove any voxels with a lower probability of being a lesion. Total volume of WMH was divided by intracranial volume, obtained through Freesurfer (https://surfer.nmr.mgh.harvard.edu/) which was then multiplied by 100 to provide a percent.

#### Resting State fMRI

Participants were instructed to fixate on a crosshair for 6 min of continuous rs-fMRI collection while keeping their eyes open. A whole brain echo-planar imaging (EPI) sequence with the following parameters was used: TR = 3,000 ms, TE = 30 ms, FOV = 24 × 24 cm^2^, 64 × 64 matrix, 42 axial slices, 3 mm slice thickness, 0.3 mm gap. MRI data were processed using default preprocessing pipeline of the Conn toolbox for MatLab (Whitfield-Gabrieli and Nieto-Castanon, 2012) implemented with SPM12 for ROI-to-ROI analysis, using the methods described by Foret et al. (2020a). Artifact Detection Tools was used for outlier detection (ART; https://www.nitrc.org/projects/artifact_detect) with default thresholds (z = 9 for global signal; 2 mm motion) and first level within-subject analysis utilized the general linear model consisting of realignment and scrubbing with a band-pass filter was set to [0.008 0.09] Hz. Denoising was performed and linear and quadratic effects of white matter and CSF BOLD time series, all first-level covariates, and rest were included as covariates. Connectivity matrices constructed between the source Posterior Cingulate Cortex (PCC) and region of interest, in this case Medial Prefrontal Cortex (MPFC), for each subject. Multivariate analysis was performed to determine the difference between PCC and DMN connectivity across subjects.

#### Magnetic-Resonance Spectroscopy

Point-RESolved Spectroscopy (PRESS) sequence (svs_se_30) to obtain cerebral metabolite ratios for ^1^H-MRS data. The following parameters were used: TE/TR = 30/3,000 ms, 80 excitations, 2,000 Hx spectral width, volume ~6 cm^3^ in the occipitoparietal gray matter including the posterior cingulate gyrus (Kaur et al., 2017). Metabolic changes in the posterior cingulate gyrus have been implicated in early stages of dementia (Herholz et al., 2002). A digital archive was saved and reviewed to maintain consistency of voxel placement. Concentrations of glutamate, mI and NAA were reported as ratios relative to creatine (Cre), a marker of energy metabolism, the most stable metabolite for use as an internal reference (Kantarci et al., 2000; Ross and Sachdev, 2004). The commercially available software, LCModel, was used to separate the metabolite resonance from the macromolecule background.

### Statistical Analyses

#### Network Analyses

Network analyses were estimated in JASP (JASP Team, 2020), which is based on the bootnet package in R (R Core Team; version 3.2.3) package qgraph (Version 1.3.3; Epskamp et al., 2012). In network analysis terminology, observed variables are referred to as nodes and relationships between observed variables as edges. Our analysis used a regularized estimation method, Extended Bayesian Information Criterion Graphical Least Absolute Shrinkage and Selection Operator (EBICglasso), which estimates partial correlations between variables and shrinks absolute weights to zero (Foygel and Drton, 2010). This method is appropriate for estimating networks when binary variables are included (van Borkulo et al., 2014). Tuning parameters were set to 0.5 and missing values were excluded pairwise from analyses to preserve as much of the sample as possible. A power analysis was performed using the netPower package in R (https://github.com/mihaiconstantin/netpaw) and revealed that, for the smallest number of individuals in a single correlation (*n* = 78 males with WMH volume), sensitivity is estimated at ~91.6% and specificity at 81.8% with <20% probability of a type I error and <10% probability of a type II error. This method estimates 100 different network models with varying degrees of sparsity. The starting value of the hyperparameter y was set to 0.5 (Foygel and Drton, 2010). We used normalized estimation of centrality measures to calculate which nodes are most central to the network (Opsahl et al., 2010). Measures of centrality for each node included betweenness (which nodes serve as bridges between other nodes in the network), closeness (relative closeness of a node to all other nodes in a network) and strength (how many direct connections a node has with other nodes).

Network graphs, also produced in JASP, are based on the R package (R Core Team; version 3.2.3) qgraph (Version 1.3.3; Epskamp et al., 2012). Positioning of the nodes was done using the Fruchterman-Reingold algorithm, which uses pseudo-random numbers to organize the network based on the strength of connections between nodes (Friedman et al., 2008, 2014; Epskamp et al., 2012, 2018).

#### Follow-Up Linear Regression Analyses

A central aim of this paper was to examine how network analysis might enhance our understanding of vulnerability to later-life cognitive decline. Thus, we conducted follow-up analyses examining the ability of the strongest nodes in the network, selected based on the methods described above where “strong” nodes were those with strength centrality measures more than 1 SD above the mean, to account for variance in current cognitive performance. Four separate linear analyses were conducted in JASP (JASP Team, 2020).

## Results

**Figure 1:**
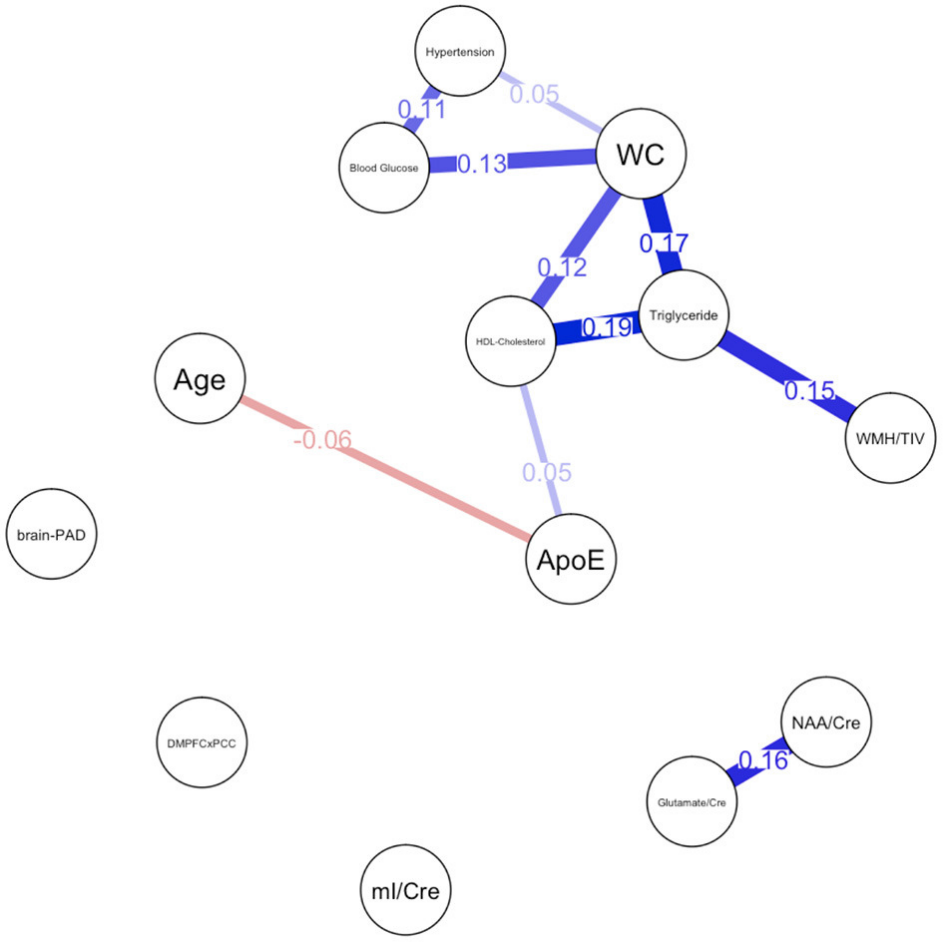
Network plot for males. Brain-PAD, Brain predicted age difference, DMNPFCxPCC, Resting State Functional Connectivity in the Default Mode Network; WC, Waist Circumference; NAA/Cre, Ratio of N-Acetylaspartate to Creatine; mI/Cre, Ratio of Myo-inositol to Creatine; WMH/TIV, White Matter Hyperintensities adjusted for total intracranial volume. Red bars indicate negative correlations and blue bars indicate positive correlations. Thicker, shorter bars indicate stronger relationships. Minimum edge strength set to 0.5 was ignored in network plots because it was larger than the absolute value of the strongest edge.

**Figure 2:**
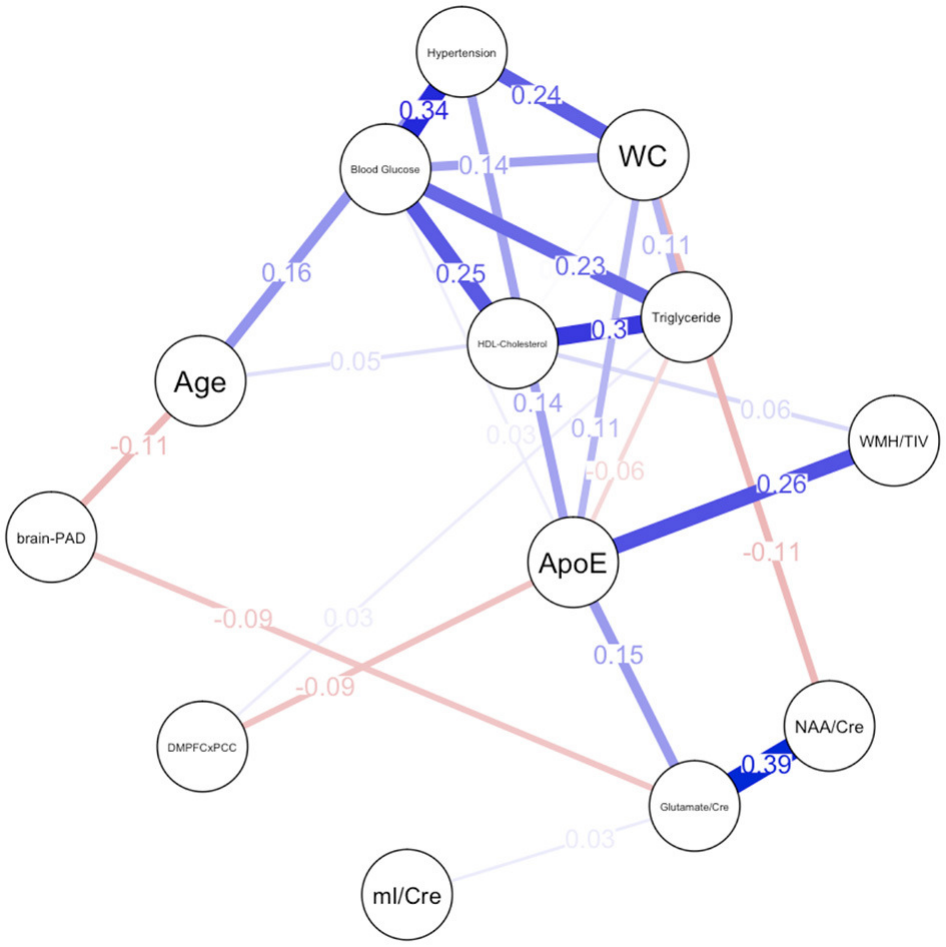
Network plot for females. Brain-PAD, Brain predicted age difference; DMNPFCxPCC, Resting State Functional Connectivity in the Default Mode Network; WC, Waist Circumference; NAA/Cre, Ratio of N-Acetylaspartate to Creatine; mI/Cre, Ratio of Myo-inositol to Creatine; WMH/TIV, White Matter Hyperintensities adjusted for total intracranial volume. Red bars indicate negative correlations and blue bars indicate positive correlations. Thicker, shorter bars indicate stronger relationships. Minimum edge strength set to 0.5 was ignored in network plots because it was larger than the absolute value of the strongest edge.

Descriptive statistical analyses (Table 1) revealed a cognitively normal middle-aged sample. As discussed in the previous section, males and females significantly differed in levels of mI as well as measures of waist circumference, HDL-cholesterol, triglycerides, blood glucose levels and functional connectivity. Sex differences in MetS variables are expected as the Alberti et al. (2009) criteria utilizes different cutoffs for HDL-cholesterol, blood pressure, and waist circumference. We have included frequencies of male and female participants meeting MetS criteria for each component in addition to the average blood pressure, blood glucose, waist circumference, triglyceride and HDL-cholesterol levels in the sample. Approximately 69% of females met criteria for elevated waist circumference vs. 47% of males, 27% met criteria for elevated triglycerides vs. 47% of males and frequencies of hypertension were similar between male and female participants. Figure 1 represents the network for males and Figure 2 represents the network for females.

## Male Network

**Figure 3:**
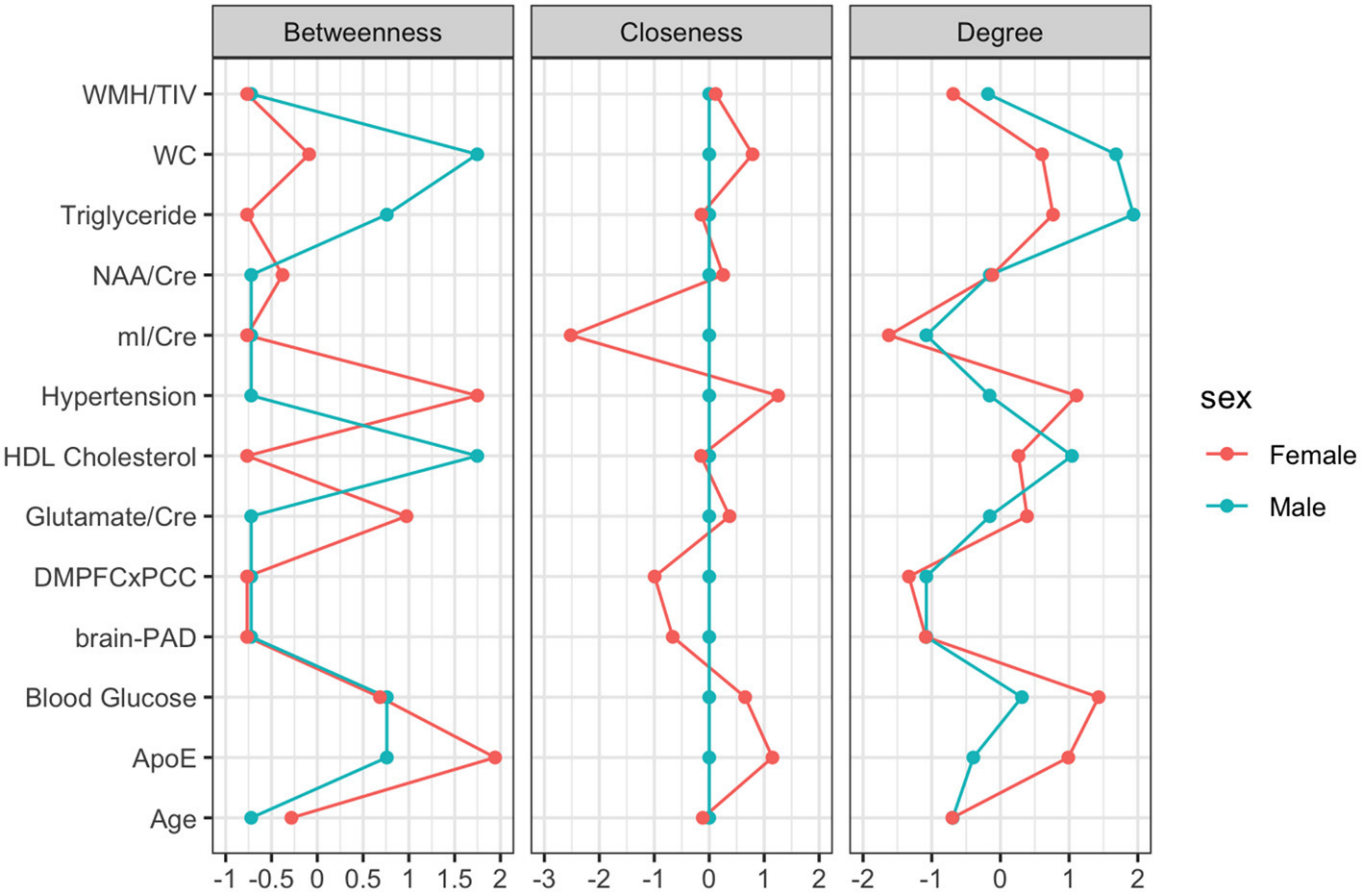
Centrality Plot for each node including betweenness (which nodes serve as bridges between other nodes in the network), closeness (relative closeness of a node to all other nodes in a network), and degree/strength (how many direct connections a node has with other nodes).

For males, ~87.2% of edges were set to zero. Centrality measures are provided in Table 2 and Figure 3. Visual examination of the graph revealed that the strongest edges were between the MetS components, particularly between plasma triglyceride levels and HDL-cholesterol as well as triglycerides and waist circumference. There was also a strong edge between levels of NAA and glutamate. According to the graph, there is a negative association between age and ApoE status for males, indicating that male ϵ4 carriers in our sample are younger overall. Nodes with measures of strength more than 1 SD above the mean for males were triglyceride levels and waist circumference, indicating that these variables might hold more information for connecting with the wider network. Measures of closeness revealed that nodes for males were not close to any other nodes in the network, which is typical of a sparsely connected network. For males, HDL-cholesterol and waist circumference had the highest measures of betweenness.

**Table 2:** Centrality measures for each node including betweenness (which nodes serve as bridges between other nodes in the network), closeness (relative closeness of a node to all other nodes in a network), and degree/strength (how many direct connections a node has with other nodes).

	**Male**			**Female**		
**Variable**	**Betweenness**	**Closeness**	**Strength**	**Betweenness**	**Closeness**	**Strength**
brain-PAD	−0.722	0.000	−1.079	−0.766	−0.664	−1.087
DMPFCxPCC	−0.722	0.000	−1.079	−0.766	−0.993	−1.333
Glutamate/Cre	−0.722	0.000	−0.154	0.975	0.370	0.388
Blood Glucose	0.760	0.000	0.312	0.684	0.654	1.430
HDL-Cholesterol	1.747	0.000	1.041	−0.766	−0.149	0.264
Hypertension	−0.722	0.000	−0.158	1.748	1.255	1.109
Triglyceride	0.760	0.000	1.937	−0.766	−0.143	0.766
WC	1.747	0.000	1.683	−0.089	0.788	0.605
NAA/Cre	−0.722	0.000	−0.154	−0.379	0.254	−0.118
WMH/TIV	−0.722	0.000	−0.181	−0.766	0.116	−0.687
Age	−0.722	0.000	−0.694	−0.283	−0.117	−0.699
ApoE	0.760	0.000	−0.394	1.942	1.150	0.989
mI/Cre	−0.722	0.000	−1.079	−0.766	−2.521	−1.625

*Brain-PAD, Brain predicted age difference; DMNPFCxPCC, Resting State Functional Connectivity in the Default Mode Network; WC, Waist Circumference; NAA, N-Acetylaspartate; mI, Myo-inositol; Cre, Creatine; WMH/TIV, White Matter Hyperintensities adjusted for total intracranial volume*.

### Female Network

For females, ~69.2% of edges were set to zero. Centrality measures are provided in Table 2 and Figure 3. Visual examination of the graph revealed several edges for females. The strongest edges were between MetS components, particularly HDL-cholesterol and triglyceride, glucose and blood pressure, as well as NAA and glutamate. ApoE status had positive and negative associations with several other variables, but in particular was positively associated with white matter hyperintensities. This indicates that ϵ4+ females in our sample may have greater white matter burden at midlife. Other edges of interest included: the positive association between ϵ4 status and waist circumference and systolic blood pressure, the negative association between NAA and waist circumference and NAA and brain-PAD, suggesting that individuals with older brains than their chronological age and higher waist circumferences have lower levels of NAA. Nodes with measures of strength more than 1 SD above the mean for females were functional connectivity in the DMN and mI, indicating that these variables might hold more information for connecting with the wider network. Measures of closeness revealed that several nodes for females were close to other nodes in the network, but functional connectivity in the DMN, hypertension, waist circumference and ApoE were all above the mean and mI was more than 1 SD above the mean closeness for the other variables. For females, variables or nodes with the highest degrees of betweenness included ApoE and hypertension.

### Follow-Up Linear Regression Analyses

The strongest male network nodes, triglycerides levels and waist circumference, analyzed as continuous variables for the purpose of the regression analysis, significantly predicted cognitive performance for male [*F*_(2, 73)_ = 3.47, *p* = 0.036] but not female [*F*_(1, 101)_ = 0.34, *p* = 0.711], participants. The strongest female network nodes, mI and DMN connectivity, predicted cognitive performance for female [*F*_(2, 99)_ = 4.65, *p* = 0.012] but not male [*F*_(2, 73)_ = 2.93, *p* = 0.060] participants. Thus, midlife cognitive performance appears sensitive to markers of metabolic dysfunction in men, while cognition appears more sensitive to brain integrity markers in women, particularly markers that are associated with risk for AD pathologies such as mI and DMN Connectivity (Voevodskaya et al., 2016, 2019).

## Discussion

In this study, we investigated relationships between cardiometabolic risk factors and brain integrity through network analysis. Network metrics suggested meaningful differences between males and females at midlife. Although the networks revealed many metrics about the relationships between brain, demographic and metabolic variables for males and females, measures of density and betweenness centrality are of the greatest interest for understanding how many links exist between variables in a network, which can be an indicator of higher risk, and which variables might bridge the relationship between other variables of interest.

### Potential Mechanisms

Overall, the findings of the present study suggest that the network for males was sparse relative to the network for females. Consistent with prior research (Pasha et al., 2018b), the network for males revealed greater interconnectedness between metabolic risk factors and white matter hyperintensities. Additionally, though one study found women to have higher levels of WMH than men overall, higher BMI was associated with higher WMH only in men (Alqarni et al., 2021), providing further support for sex differences in the relationship between metabolic risk factors and WMH. HDL cholesterol and waist circumference appear to be of particular importance for males as this node had the highest level of betweenness. Low HDL-cholesterol and elevated triglyceride levels, which also had a high degree of strength centrality in the network, have been associated with increased risk for dementia only in men in another study (Ancelin et al., 2013). Waist circumference has emerged as an important predictor of cardiovascular disease markers, such as elevated C-reactive protein, over the other MetS components for both sexes (Nakamura et al., 2008; Cheong et al., 2015), though our findings suggest that this effect may be more robust for males.

For females, the network had a higher measure of density and indicated many relationships between metabolic risk factors, brain integrity and genetic status at midlife. Though strong edges were visible between MetS components in females as in males, relationships between WMH and metabolic syndrome components were weak. This finding is consistent with the findings discussed previously on sex differences in white matter burden (Pasha et al., 2018b; Alqarni et al., 2021). Most notable was the centrality of ApoE and age in the graphical model of the female network. Most notable was the centrality of ApoE and age in the graphical model of the female network. The prevalence of ϵ4+ individuals in our sample is ~15%, which is consistent with the general population (Heffernan et al., 2016). This, unfortunately, results in an unbalanced sample for the network analysis. It would be interesting to re-examine the female network in sample with a more balanced ratio of ϵ4+ and ϵ4- individuals, to see if it remains stable. Though our findings are somewhat limited by the small sample of ϵ4+ individuals, our results suggest that age and genetics may play an important role in driving brain-metabolic health relationships in midlife, which is consistent with prior literature (Plassman et al., 2010). ApoE ϵ4 status has been shown to have larger impact on memory performance and hippocampal atrophy in women than in men (Azad et al., 2007), and this network suggests that midlife cardiovascular mechanisms might be responsible for this relationship. This finding is unsurprising, as ApoE ϵ4 status conveys greater risk of neurocognitive and cardiovascular disease for females (Mortensen and Høgh, 2001; Riedel et al., 2016), and sex has been found to moderate associations between amyloid burden, ϵ4 status and functional connectivity in the DMN (Damoiseaux et al., 2012; Caldwell et al., 2019). Biological changes occurring during the menopausal transition may lead to additional vulnerabilities in cardiovascular and metabolic health (Gordon et al., 1978; El Khoudary et al., 2020). Since the average age at natural menopause in the United States is around 52.6 years (Reynolds and Obermeyer, 2005), the age range in our sample could be capturing women with variable hormonal profiles that encompass premenopausal, perimenopausal, and postmenopausal women. Recent literature has suggested that perimenopause, in particular, could drive changes in brain integrity and metabolic processes (Brinton et al., 2015; Palla et al., 2020). Reproducibility of self-reported menopausal status varies (Paganini-Hill and Ross, 1982; Horwitz and Yu, 1985; Colditz et al., 1987; den Tonkelaar, 1997; Rödström et al., 2005). However, it is important for these issues to continue to be explored further by measuring the endogenous levels of relevant sex hormones (Wildman et al., 2008) and documenting the use of HRT, as many sex differences in cardiovascular disease are attributed to protective effects of estrogen (Stanhewicz et al., 2018; Peters et al., 2019; Rodgers et al., 2019). Due to the small number of participants on HRT, we were unable to examine any potential effects of medication. Future studies with a larger number of participants on HRT could examine the role of either testosterone or estrogen therapies on relationships between brain integrity and metabolic function using a similar statistical technique to our analysis as research studies on HRT to protect against neurocognitive decline have shown mixed findings (LeBlanc et al., 2001; Wu et al., 2020).

Somewhat challenging to interpret is the high level of betweenness of systolic blood pressure for females. Hypertension in midlife has been associated with increased risk for dementia among women but not men (Gilsanz et al., 2017). Another study has shown that midlife hypercholesterolemia and hypertension convey risk for dementia in both men and women (Azad et al., 2007). Even though our networks demonstrate that there are sex differences in the degree of impact of these risk factors on brain health, it is unclear if hypertension acts as the driving force for other MetS risk factors. Unlike ApoE, the betweenness of hypertension appears to be driven more by its relationship with other MetS risk factors rather than a position between brain integrity and MetS. Females in our sample who meet MetS criteria for elevated systolic blood pressure appear to be older and more likely to be ϵ4 carriers (Figure 2), which re-emphasizes the significance of ϵ4 status in the network.

In both males and females, glutamate and NAA were positively related to one another. Concentrations of these neural metabolites have been correlated in other research, with some hypothesizing that NAA can be converted to glutamate when supplies are low (Clark et al., 2006). Ultimately, these ^1^ H MRS findings are difficult to untangle and only suggest that our sample is relatively healthy without notable levels of pathology. However, an edge between NAA and waist circumference was visible only in females, such that females meeting MetS criteria for elevated waist circumference had lower levels of NAA. Relationships between female neuronal viability and waist circumference are not widely studied, but previous research has found that elevated BMI and subclinical atherosclerosis is associated with lower levels of NAA in the anterior (Gazdzinski et al., 2010) and posterior cingulate cortex (Haley et al., 2010b). Additionally, NAA was associated with brain-PAD in females, such that women with greater brain-age gaps might have poorer neuronal viability. Though application of brain-aging algorithms in a midlife population is still relatively novel, previous literature using a similar technique to analyze brain-PAD (brainageR, https://github.com/james-cole/brainageR) has shown that accelerated brain aging may be observable in midlife women in relation to lifestyle factors which impact hormone levels, in this case number of childbirths. Further, their findings are consistent with observed patterns of parity and risk of AD, where increased number of childbirths conveys lower risk of neurocognitive decline (de Lange et al., 2019). This provides additional evidence that brain-PAD at midlife could relate to later life outcomes and that female populations experience hormonal changes throughout their life that convey unique risk factors for neurocognitive decline. Myo-inositol provided the least information of any node in the network, which is surprising as myo-inositol and the other brain variables selected are considered preclinical markers of AD, and elevations have been found in asymptomatic individuals with cardiometabolic risk (Kantarci et al., 2000; Haley et al., 2010a; Voevodskaya et al., 2016, 2019).

The application of network modeling in this study is novel and significant in that graph-theory techniques can contribute unique information to cognitive risk assessment about the interconnectedness and organization of relationships among risk factors, over and above the measured levels of physiological variables of neurobiological significance. Research applying network modeling to psychopathology suggests that a more tightly connected network is riskier because “activation” of one symptom can spread to others (Borsboom and Cramer, 2013). This seems even more likely with biological systems as there are mechanistic relationships between nodes in our network. Relationships in the male body-brain network suggest that males have greater vulnerability than females to cerebrovascular lesions under conditions of metabolic syndrome. The female picture appears to be more complicated. Our network analyses suggest direct relationships between ϵ4 status and metabolic risk factors in women, such as waist circumference and systolic blood pressure, supporting previous research on the role of ApoE in females with cardiovascular disease (Sampedro et al., 2015; Riedel et al., 2016). As mentioned, women have higher incidence of AD (Zhao et al., 2016; Andrew and Tierney, 2018; Beam et al., 2018; Buckley et al., 2019) and cardiovascular risk factors have been suggested as a mechanism for this disparity (Volgman et al., 2019). Our follow-up linear regression analyses demonstrate the ability of the strongest nodes in the sex-specific body-brain network models to better account for variance in current cognitive performance of their respective sex, even in midlife when cognitive function is relatively preserved. These findings further support the utility of the network analysis method to identify variables which convey unique vulnerability for neurocognitive decline in male and female populations. In midlife, the constructed sex-specific networks also provide valuable information about mechanisms of brain vulnerability in at-risk populations, before cognitive function is significantly impaired, by simultaneously examining the effects of multiple physiological variables on each other as well as on brain and cognitive function.

Our study assessed sex, not gender identity, so our findings may or may not generalize to transgender men and women. As our work stands, it is unclear whether observed sex differences are a result of biological mechanisms based on genotype, hormone levels or sociocultural experiences of sex and gender. However, these observed differences between midlife males and females suggest a personalized medical approach which takes sex into consideration as key to early identification and management of modifiable risk factors for cognitive aging.

## Conclusions

Our findings support prior research on sex differences in relationships between cardiometabolic risk, genetics and brain integrity and provide further support for a personalized medicine approach which takes sex into consideration. Network analysis has the additional benefit of untangling complex mechanisms by allowing researchers and clinicians to consider multiple variables at once. The network for females suggests an important influence of genetic status on metabolic risk and brain integrity and may warrant additional attention when presenting clinically with any of these risk factors, which may be modifiable with appropriate pharmaceutical or behavioral intervention.

## Data Availability Statement

The data analyzed in this study is subject to the following licenses/restrictions: Data and code available on request. Requests to access these datasets should be directed to haley@austin.utexas.edu.

## Ethics Statement

The studies involving human participants were reviewed and approved by Institutional Review Board University of Texas at Austin. The patients/participants provided their written informed consent to participate in this study.

## Author Contributions

JF and AH: conception and study design. AH and HT: resources, project administration, and funding acquisition. JF, MD, and JC: statistical analysis. JF, MD, MC, and AH: interpretation of results. JF, MD, DG, MC, HT, and AH: drafting the manuscript work or revising it critically for important intellectual content. All authors: approval of final version to be published and agreement to be accountable for the integrity and accuracy of all aspects of the work.

## Conflict of Interest

The authors declare that the research was conducted in the absence of any commercial or financial relationships that could be construed as a potential conflict of interest.
